# Tumor Angiogenesis as a Target for Dietary Cancer Prevention

**DOI:** 10.1155/2012/879623

**Published:** 2011-09-29

**Authors:** William W. Li, Vincent W. Li, Michelle Hutnik, Albert S. Chiou

**Affiliations:** The Angiogenesis Foundation, One Broadway, 14th Floor, Cambridge, MA 02142, USA

## Abstract

Between 2000 and 2050, the number of new cancer patients diagnosed annually is expected to double, with an accompanying increase in treatment costs of more than $80 billion over just the next decade. Efficacious strategies for cancer prevention will therefore be vital for improving patients' quality of life and reducing healthcare costs. Judah Folkman first proposed antiangiogenesis as a strategy for preventing dormant microtumors from progressing to invasive cancer. Although antiangiogenic drugs are now available for many advanced malignancies (colorectal, lung, breast, kidney, liver, brain, thyroid, neuroendocrine, multiple myeloma, myelodysplastic syndrome), cost and toxicity considerations preclude their broad use for cancer prevention. Potent antiangiogenic molecules have now been identified in dietary sources, suggesting that a rationally designed antiangiogenic diet could provide a safe, widely available, and novel strategy for preventing cancer. This paper presents the scientific, epidemiologic, and clinical evidence supporting the role of an antiangiogenic diet for cancer prevention.

## 1. Introduction

Cancer now affects as many as 24 million people worldwide, and results in over six million deaths each year [1]. In the United States, men and women have a 43% and 38% chance, respectively, of being diagnosed with any type of cancer during their lifetime [2]. Despite advances in the early detection of cancer, most malignancies are still diagnosed and treated at advanced stages, with a limited range of therapeutic options and poor overall survival. Simultaneously, cancer treatment costs are escalating, from $125 billion annually in the US in 2010 to a projected $207 billion by 2020 [3]. Cancer prevention is, thus, a key opportunity for managing the cancer pandemic. Unlike interventional approaches delivered to patients diagnosed with advanced cancer, preventative strategies must be suitable for healthy individuals and have low systemic toxicity, inhibiting microscopic tumor growth with minimal adverse effects on healthy tissues [4].

It is now well established that solid tumor growth is dependent upon angiogenesis, the growth of new blood vessels [5–10]. During early stages of tumorigenesis, the induction of angiogenesis by cancer cells is a critical event separating the preinvasive and dormant form of cancer from the invasive and metastatic phases of malignant growth. Multiple studies have demonstrated that the degree of tumor vascularity correlates positively with disease stage, the likelihood of metastases, and cancer recurrence [11, 12]. Angiogenesis also plays a role in hematogenous malignancies, such as leukemia, lymphoma, and multiple myeloma, as well as in premalignant myelodysplastic syndromes [13–17]. In these pathologies, vascular endothelial cells sustain and promote malignant cell growth by secreting paracrine survival factors [18, 19]. 

Antiangiogenic therapy has been validated as an effective cancer treatment strategy for a growing number of cancer types, including colorectal, renal, liver, lung, brain, pancreatic neuroendocrine tumors (NET), gastrointestinal stromal tumors (GIST), multiple myeloma, and myelodysplastic syndrome [20]. More than 120 novel antiangiogenic agents are in clinical, trials [20–22]. Importantly, a growing body of preclinical, clinical and epidemiological data is demonstrating that angiogenesis inhibition can be applied for achieving cancer prevention [23, 24]. This paper presents the scientific and clinical evidence supporting antiangiogenesis as a rational strategy for the prevention of cancer, exploiting factors that are naturally present in dietary sources.

## 2. The Physiological State of Angiogenesis Regulation

The human body contains 60,000 miles of blood vessels, including 19 billion capillaries. All normal cells in the body are located no further than 100–200 *μ*m from the nearest capillary, the diffusion limit of oxygen [25]. Capillaries not only deliver oxygen and micronutrients to tissues, but the endothelial cells comprising them secrete paracrine growth and survival signals that influence adjacent nonvascular cells [18]. Under physiological conditions, the rate of cell proliferation is balanced with the rate of cell death (apoptosis), so there is no net tissue growth. Expansion of tissue mass requires angiogenesis to support increased metabolic demand [9]. In normal healthy adults, angiogenesis is constitutively suppressed except for brief bursts during the female reproductive cycle (endometrial regeneration, corpus luteum formation), pregnancy (placentation), and wound healing (granulation) [26–30]. The physiological state is thus maintained in a constitutive state of suppressed angiogenesis by endogenous inhibitory mechanisms opposing the action of angiogenic growth and other stimulating factors.

### 2.1. Angiogenic Growth Factors

More than 30 endogenous molecules have been identified as angiogenic factors (Table 1). These share the ability to stimulate neovascularization *in vivo* and induce endothelial proliferation, migration, or capillary tube formation *in vitro*. Basic fibroblast growth factor (bFGF or FGF2) was the first angiogenic factor to be identified from a tumor extract, but vascular endothelial growth factor/vascular permeability factor (VEGF/VPF) is the best studied [30]. VEGF is a potent endothelial mitogen that increases vascular permeability, and also induces Bcl-2, promoting vascular survival [31–34]. VEGF is expressed by all human tumors studied and its receptors (VEGFR-1, -R2, -R3 and -R4) are expressed selectively on angiogenic endothelial cells [35]. Placental growth factor (PlGF) plays a specific role in pathological neovascularization by recruiting bone marrow-derived vascular stem cells to disease sites [36, 37]. Other factors include platelet-derived growth factor (PDGF), platelet-derived endothelial cell growth factor (PD-ECGF), interleukin-3 (IL-3), interleukin-8 (IL-8), transforming growth factor-*β* (TGF*β*), and tumor necrosis factor-alpha (TNF*α*) [38, 39]. Other angiogenic factors are neuregulin, a ligand for the ErbB receptor, and keratinocyte growth factor (KGF or FGF-7) [40, 41]. Angiogenic factors are observed at low levels or undetectable in the circulation in normal, healthy subjects. By contrast, markedly elevated levels of factors such as bFGF, VEGF, and PD-ECGF are present in the serum, urine, and cerebrospinal fluid of cancer patients [42, 43].

### 2.2. Physiological Inhibition of Angiogenesis

Angiogenesis inhibitory activity was first discovered in studies of cartilage, a naturally avascular tissue [44]. Numerous endogenous antiangiogenic molecules have subsequently been identified, including troponin-1, tissue inhibitors of matrix metalloproteinases (TIMPs), chondromodulin I, connective tissue-growth factor (CTGF), decorin, metastatin, pigment epithelium-derived factor (PEDF) thrombospondin-1 and -2, interferons, tetrahydrocortisol-S, platelet factor-4, and protamine [45–60]. Other inhibitors, such as canstatin, tumstatin, and arresten, are present in the basement membrane surrounding established blood vessels [61–63].

A separate and distinct class of inhibitors is comprised of proteolytic fragments derived from cleaved larger molecules. Angiostatin is an internal fragment of plasminogen and specifically inhibits endothelial cell proliferation [64]. Enzymes such as macrophage-derived elastase and serine proteases generate angiostatin or angiostatin-like fragments [65, 66]. Endostatin, a 20-kDa fragment of collagen XVIII, is a specific angiogenesis inhibitor that induces endothelial apoptosis [67, 68]. Both angiostatin and endostatin were discovered in the serum of tumor-bearing experimental mice, suggesting that tumor-associated protease activity generates these inhibitors. Removal of the primary tumor led to a marked decline in serum angiostatin and endostatin, followed by rapid angiogenic growth of metastatic lesions [9, 69]. Endostatin is present at a low circulating level in normal subjects [70]. Collectively, these endogenous angiogenesis inhibitors play a dominant role in suppressing angiogenesis in health and contribute to tumor dormancy (Table 2).

### 2.3. Balance and Imbalance of Angiogenesis

Vascular growth is physiologically governed by a homeostatic balance between positive and negative angiogenesis regulators, so that neovascularization is normally suppressed [71]. Vascular proliferation occurs when angiogenic growth factor production is upregulated, or when expression of endogenous inhibitors is downregulated, or when both events occur simultaneously [72–74]. The genetic regulators of angiogenesis are closely related to tumor growth promotion and suppression (Table 3). Gene knockout studies in mice have shown that Id1 and Id3, peptides that control cell differentiation by interfering with DNA binding of transcription factors, are required for normal vascular formation and induction of angiogenesis in tumor-bearing animals [75]. The activated forms of the oncogenes H-*ras*, v-*raf*, c-*myc*, c-*src*, Her-2/neu, and p73 are associated with cellular production of VEGF as well as tumorigenesis [76–83]. Several tumor-suppressor genes regulate angiogenesis inhibition, including p53, Rb, vHL, phosphatase and tensin homolog (PTEN), and *trk *B [84] Wild-type p53 controls expression of the angiogenesis inhibitor thrombospondin and decreases tumor neovascularization; mutant p53 leads to the opposite effect [73]. The retinoblastoma (Rb) gene and the von Hippel-Lindau (vHL) gene both downregulate VEGF expression; their mutation leads to VEGF production, angiogenesis, and tumor growth [85, 86].

### 2.4. The Avascular Dormant Phase of Cancer

Microscopic cancer cells are commonly present in the healthy adult, the result of errors during replication of 60–90 trillion cells. To acquire sustenance, the incipient tumors (60–80 cells) may migrate toward existing host vessels, a process known as vessel cooption, but their growth remains limited [87–89]. Tumors are capable of growth to approx 0.5 mm^3^ in diameter (10,000,000 cells) before reaching a steady state of growth. Beyond this size, their metabolic demands exceed the supply of oxygen and nutrients obtained by passive diffusion from nearby blood vessels. This state corresponds to carcinoma *in situ*, and the rate of tumor cell proliferation is balanced by apoptosis [90]. Such microscopic tumors may exist for years without clinical detection.

Autopsy studies have shown that these microscopic cancers are present in the breasts of up to 40% of women between the ages of 40 and 50 years, and in 50% of prostates in men between 50 and 60 years. By age 70, microscopic cancers are detected in the thyroids of virtually everyone. Most of these tumors never become clinically significant, leading to the concept of “cancer without disease” as a normal state during aging. Physiological angiogenesis inhibition is regarded as one of the mechanisms that prevent microscopic cancers from converting to a malignant phenotype.

### 2.5. The “Switch” to the Angiogenic Phenotype during Multistep Tumorigenesis

**Figure 1:**
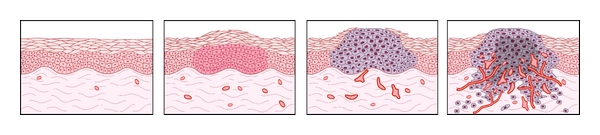
The switch to the angiogenic phenotype occurs during multistage tumorigenesis. As malignancy develops, cells progress from a prevascular stage (normal to early hyperplasia) to a vascular stage (late hyperplasia to dysplasia to invasive carcinoma). Angiogenesis becomes clearly evident during dysplasia and is critical for further growth. Targeting tumor angiogenesis may be a novel strategy for preventing cancer. (Reprinted by permission from the Angiogenesis Foundation. Copyright 2011 by The Angiogenesis Foundation. All rights reserved).

To expand beyond the limits of the preexisting vascular supply, tumors recruit new blood vessels from surrounding vessels, an event known as the “switch” to the angiogenic phenotype [91] (see Figure 1). Three classic studies employing transgenic mice have delineated this switch as normal cells undergo the transition from normalcy to hyperplasia to dysplasia, and finally to frank carcinoma.

In a model of spontaneous *β*-islet cell tumor formation, Rip1-Tag2 transgenic mice selectively express the SV40 T-antigen oncogene in their insulin producing *β* cells and undergo a predictable sequence of multistep tumorigenesis [71, 92, 93]. The transformed *β*-cells are localized to approx 400 islets in the pancreas, of which 100% express the oncogene. Over time, 50–70% of these islets become hyperplastic nodules. A distinct angiogenic stage occurs at 6-7 wk of age between the hyperplastic stage and the time at which subset islets become invasive carcinomas at 12–16 weeks. The angiogenic capacity of these lesions is observed as visible intense tumor vascularization, accompanied by induction of capillary sprouting, endothelial proliferation, and a starburst-like convergence of capillaries when islets are harvested *in vivo* and cocultured with endothelial cells *in vitro*. Importantly, nonangiogenic islets are unable to grow beyond 0.6–0.8 mm^3^ in size, whereas the small subset of angiogenic islets can expand into a lethal tumor burden [94].

A second study utilized the bovine papillomavirus oncogene in a transgenic mouse model of dermal fibrosarcoma [95, 96]. Distinct stages of tumorigenesis are observed, from normal cells to a proliferative hyperplastic stage (mild and aggressive fibromas) to neoplasia (fibrosarcoma). The preneoplastic fibromas grow horizontally within the dermis as thin avascular lesions, and the fibrosarcomas are expansile and densely vascularized. Angiogenesis is first observed during the late preneoplastic stage (aggressive fibroma) and sustained until death of the animals by fibrosarcoma. Aggressive fibromas and fibrosarcomas secrete bFGF. By contrast, bFGF is not secreted by normal cells or by mild fibromas.

A third study involved K14-HPV16 transgenic mice in which the human papillomavirus (HPV) type 16 oncogene is targeted to expression in basal cells of the epidermis by regulatory elements of the human keratin-14 promoter [97]. These basal keratinocytes undergo sequential changes from normal cells (no vascularization) to hyperplasia (mild vascularization from the underlying dermis) to dysplasia (abundant vessels under the basement membrane in close apposition to aberrant keratinocytes) to squamous cell carcinoma (intense angiogenesis breaching the basement membrane into the tumor). In hyperplasia, dysplasia, and at the invading cancer front, angiogenesis is associated with mast cell infiltration and degranulation [98]. Mast cells contain numerous angiogenic stimulators in their secretory granules, such as the serine protease MCP-4, VEGF, bFGF, TGF*β*, TNF*α*, and IL-8 [99, 100]. In dysplasia and carcinoma, tissue expression of VEGF was increased, correlating to increased tumor vessel density [101].

Together, these data demonstrate that angiogenesis is a discrete, genetically regulated and rate-limiting step during multistep tumorigenesis, that the transition from prevascular to vascular phase is accompanied by the production and release of one or more angiogenic growth factors, and that host inflammatory cells may amplify the angiogenic switch by contributing additional stimuli.

### 2.6. The Vascular Phase of Cancer

The onset of angiogenesis precedes an exponential phase of tumor growth accompanied by local organ invasion. The velocity of angiogenic capillary growth ranges from 0.223 to 0.8 mm/day [102–104]. Studies of avascular tumor explants placed in the anterior chamber of the eye show that once new vessels reach the explant, tumors can expand 16,000-fold in size in 2 wk [105]. During this expansion, cancer cells grow as a cuff around each new microvessel with a thickness of 50–200 *μ*m. In this configuration, one endothelial cell supports the metabolic needs of 5–100 cancer cells [106, 107]. Eventually, invading blood vessels occupy 1.5% of the tumor volume [108]. Tumor angiogenesis also facilitates cancer metastases by allowing cells to exit through the neovascular network into the systemic circulation [109]. Elegant studies of mammary carcinomas in mice have shown that a 1 cm tumor sheds up to 4 × 10 − 6 malignant cells into the circulation every 24 h [110].

## 3. Targeting Tumor Angiogenesis for Cancer Prevention

The concept of “antiangiogenesis” was first proposed in 1971 by Judah Folkman, who hypothesized that inhibition of neovascularization at an early stage of cancer development could prevent tumor growth and metastases and maintain tumor dormancy [7]. A vast literature establishes that angiogenesis inhibition is an effective strategy to restrict cancer growth in animal models bearing a wide variety of cancers [8, 111]. To date, more than 300 angiogenesis-inhibitory molecules have been identified as potential drug candidates, including many natural and synthetic chemical entities (reviewed in *55*). Selective targeting of angiogenic blood vessels is possible as a result of differential proliferation rates between normal and tumor-associated endothelium. The normal vasculature is highly quiescent, with only one in every 10,000 endothelial cells dividing at any given time, and a physiological doubling time ranging from 47 to 20,000 days [112–114]. In contrast, the doubling rate for tumor endothelium is 2–13 days. Thus, antiangiogenic agents are selective in inhibiting proliferating tumor vasculature, but do not affect normal blood vessels. 

### 3.1. Targets of Tumor Angiogenesis

Specific molecular and cellular targets have been identified for tumor angiogenesis. These include targets present during the orderly events characterizing new blood vessel growth [115]. These include (1) angiogenic growth factor production, release, and receptor activation, (2) degradation of vascular basement membrane, (3) endothelial proliferation, migration, and survival, (4) blood vessel sprouting and invasion, (5) tubular morphogenesis, (6) arterial-venous patterning, (7) vascular maturation, and (8) recruitment of endothelial stem cells.

### 3.2. Clinical Principles of Antiangiogenic Therapy

The clinical development of antiangiogenic therapy began in the late 1980s. The first successful treatment of a vascular tumor (pulmonary hemangiomatosis) occurred in 1989 using interferon-*α* 2a as an antiendothelial agent [116]. The first drug to enter formal clinical trials as an angiogenesis inhibitor was TNP-470 in 1992 [117]. Since 2004, more than 14 different antiangiogenic agents have been demonstrated to be efficacious in treating solid and hematogenous cancers, primarily in the setting of advanced disease (see Table 4). To date, most agents that are specific or selective for angiogenesis are well tolerated in humans, with fewer serious (Grade 3 and 4) toxicities observed in their clinical trials when compared to cytotoxic chemotherapy drugs [118–120]. Because only proliferating endothelium is targeted, the traditional side effects of chemotherapy, such as leukopenia, alopecia, and mucositis, are rarely observed. With some antiangiogenic agents, a maximum tolerated dose (MTD) cannot be determined [70]. This has led some clinical investigations to incorporate pharmacodynamic techniques for determining the optimal biological dose (OBD) of agents in clinical trials.

Collectively, translational research studies have shown that overall disease burden is important to consider in the evaluation of the clinical effects of any antiangiogenic agent. Advanced cancers contain well-established, extensive vascular networks that may respond minimally to angiogenesis inhibitors. Vascular destructive agents, also known as vascular targeting agents, may be required to achieve a clinically significant effect on tumor perfusion [121–123]. Indeed, most preclinical studies of angiogenesis inhibitors demonstrate drug efficacy in the setting of incipient disease (prevention) or small tumors (early intervention). Clinical trials of the same agents, however, have often enrolled patients with advanced, metastastic, and heavily pretreated disease, perhaps explaining differences between mice studies and the results of human trials [124]. Antiangiogenic therapy in the adjuvant setting to suppress minimal residual disease, or as an intervention for early-stage disease or cancer prevention, has been proposed as the scenario of the greatest clinical benefit using angiogenesis inhibitors [125].

The remainder of this paper is devoted to discussing the potential for angiogenesis inhibitors for prevention of cancer.

## 4. Early Intervention and Cancer Prevention

### 4.1. Early Intervention

Angiogenesis inhibition offers an opportunity to interrupt an early, rate-limiting step in tumorigenesis [126, 127]. Suppression of pathological blood-vessel growth prevents early tumors from progressing to the malignant phenotype. Clinical correlates to preinvasive angiogenic lesions are commonly encountered in breast (ductal carcinoma *in situ*—DCIS), cervix (cervical intraepithelial neoplasia—CIN), skin (actinic keratosis), oropharynx (late Barrett's esophagus), lung (squamous metaplasia with dysplasia in bronchial mucosa), colon (premalignant adenoma), and prostate (high-grade prostate intraepithelial neoplasia, HGPIN) [128–133]. Microscopic metastases are also present in many cancer patients who are undergoing tumor resection with curative intent. For example, 25% of colon cancer patients eventually develop hepatic metastases after primary tumor resection emerging from preangiogenic lesions that were present at the time of surgery [134–136]. By suppressing tumor neovascularization at subclinical stages, tumor progression and metastatic growth may be halted.

### 4.2. Superiority of Early Therapy and Sustained Angiogenesis Suppression

Animal studies have demonstrated that early administration of angiogenesis inhibitors is highly efficacious. The drug TNP-470 (*O*-[chloroacetyl-carbonyl] fumagillol) is a potent antiangiogenic analog of the antibiotic fumagillin [124, 137]. A rat model of liver metastasis using K12/TRb rat colon adenocarcinoma cells was employed to study the differential efficacy of early, early prolonged, or delayed administration of TNP-470 (15 mg/kg) on metastatic burden and survival [134]. Treatment initiated at d1 after tumor inoculation (early intervention) and maintained for 28d (prolonged therapy) led to a 46% reduction in liver metastases and improved survival time compared to controls (*P* = 0.011). Another study showed a superior reduction in metastases with early (d 0–6) compared to delayed (d 7–13) TNP-470 treatment in rabbits bearing VX2 carcinoma [138].

The effect of TNP-470 on subclinical disease has also been elegantly studied by Shusterman and colleagues [139]. In the first study, xenografts of human neuroblastoma-derived CHP-134 were implanted into athymic (*nu/nu*) mice, with initiation of antiangiogenic treatment 12 h following grafting (early primary tumor model). Treated tumors were reduced by 90%, compared to control animals. The second study involved administration of TNP-470 12 h following tail vein injection of CHP-134 cells into SCID/Beige mice (metastatic model). Autopsy of saline-treated control mice showed neuroblastoma deposits in the kidney, liver, adrenal gland, and ovaries in 75% of subjects, whereas TNP-470-treated mice showed no evidence of metastases. A third study evaluated TNP-470 effects in mice whose tumors were initially 0.35 mm^3^, but then became difficult to palpate following 10 d of cyclophosphamide treatment (minimal residual disease model). TNP-470 was then administered subcutaneously. Tumor growth was suppressed in the TNP-470-treated group by 82%, compared to saline-treated controls. Histopathological analyses showed increased apoptosis by TdT-mediated nick-end labeling (TUNEL) assay in treated animals, but no with difference in tumor cell proliferation by Ki-67 assessment [90, 139]. These data demonstrate the importance of timing in antiangiogenic therapy and its efficacy in subclinical disease.

### 4.3. Evidence for Antiangiogenic Cancer Prevention

Cancer chemoprevention is defined as the use of pharmacological, natural, or dietary agents to inhibit the development of invasive cancer by blocking DNA damage caused by carcinogens or by arresting the progression of premalignant cells after damage has already occurred [4]. Angiogenesis inhibition blocks carcinogenesis by preventing progression to the invasive phenotype [111, 140, 141]. A number of well-known chemopreventive agents have antiangiogenic properties *in vivo* and *in vitro* (see Table 5). These include retinoids, vitamin D3, tamoxifen, oltipraz, curcumin, linoleic acid, ellagic acid, selenium, *α*-difluoromethylornithine (DFMO), *N*-acetyl-*l*-cysteine (NAC), catechins, and celecoxib [142–151]. Classical angiogenesis assay systems, such as the chorioallantoic membrane assay (CAM), the corneal micropocket assay, and modified rat aortic ring assay, have been used to screen for biological activity of established chemopreventive agents [152, 153]. Known angiogenesis inhibitors such as endostatin have also been shown to suppress carcinogen-induced tumor development in rodent models [154]. The antiangiogenic properties of select chemopreventive molecules shall be discussed.

### 4.4. Antiangiogenic Factors in Dietary Sources 

#### 4.4.1. Green Tea Catechins

After water, tea is the second most popular liquid in the world, and its consumption is linked with a decreased risk of colon, prostate, lung, esophageal, and other cancers [155–157]. Laboratory studies have demonstrated that green tea and its catechins prevent mutagenesis, tumorigenesis, cancer invasion and metastases, and angiogenesis [158–163]. Polyphenol catechins in tea, predominantly flavanols, possess chemopreventive and antiangiogenic activity. Epigallocatechin-3-gallate (EGCG) is a potent tea flavonoid that specifically inhibits endothelial cell proliferation stimulated by bFGF and induces avascular zones in the chick CAM assay. Mice that consume 1.25% green tea (human equivalent of drinking 2-3 cups of tea/day) show inhibition of VEGF-stimulated corneal neovascularization by as much as 70% and reduction of tumor cell invasion by 50% [163]. Green tea solution 0.6% administered to mice as the sole source of drinking fluid results in less tissue VEGF expression seen by immunostaining and lower microvessel density in lung adenomas, as well as significantly fewer tumors induced by the NNK carcinogen [162].

The molecular mechanism of EGCG is the result of its inhibition of urokinase and two gelatinases (MMP-2 and -9) involved in vascular as well as tumor invasion [164, 165]. The MMP inhibitory activity is independent of zinc or calcium binding by EGCG. High doses of EGCG induce apoptosis when topically applied to SKH-1 hairless mice bearing UVB-induced squamous cell carcinomas [166]. Clinical trials are underway in Western and Asian nations to study the chemopreventive potential of green tea for oral, prostate, skin, and other cancers.

Preliminary data from these trials suggest clinical efficacy. An Italian study involving men with high-grade prostate intraepithelial neoplasia (HGPIN) demonstrated a protective effect from consuming daily green tea catechins over the course of a single year [167]. The double-blind, placebo-control study randomized sixty men with HGPIN into a treatment arm receiving 600 mg of purified green tea catechins (equivalent to 2-3 cups of tea/day) and a placebo arm. The men were followed for one year, at which point they underwent prostate mapping via core needle biopsies. Based on those biopsy results, 30% of the placebo group progressed to prostate cancer, while the treatment arm demonstrated an impressively low progression rate of 3%. Similarly, a Japanese interventional study of patients with recently removed colonic adenomas demonstrated that participants drinking an average of 12 cups equivalent of green tea polyphenols, obtained through both purified extracts and whole tea, had a 50% reduction in risk of adenoma recurrence compared to patients who drank an average of six cups of green tea [168]. Chemopreventive effects in humans have also been observed through experimental trials involving the treatment of precancerous oral and cervical lesions [169, 170].

Polyphenon E, an extract from green tea leaves containing a defined mixture of polyphenolic catechins, is in clinical trials for prostate, bladder, esophageal, lung, head and neck cancers, and leukemia. In topical form, Polyphenon E 15% ointment was approved by the U.S. FDA in 2006 as a treatment for external genital warts, which is considered an angiogenic neoplasm and a precursor to cervical cancer [171].

#### 4.4.2. Genistein

Genistein (4′,5,7-trihydroxyisoflavone), an isoflavonoid found in soybeans, has both chemopreventive and antiangiogenic activity. It suppresses carcinogenesis in a variety of animal models of mammary and prostate carcinoma following oral and parenteral administration [172–174]. Multiple antitumor mechanisms of action have been identified, including angiogenesis inhibition, induction of apoptosis, G2 cell cycle arrest, inhibition of c-*fos *expression and NF-*κ*B activation, modulation of sex steroid receptors and growth factor signaling pathways [175–177]. Genistein inhibits angiogenesis by the following mechanisms: inhibition of bFGF- and VEGF-driven endothelial cell proliferation, migration, and tube formation; inhibition of extracellular matrix degradation by suppression of bFGF-induced endothelial production of plasminogen activator (PA) and plasminogen activator inhibitor (PAI); and suppression of receptor tyrosine kinase activity for VEGF, EGF, and PDGF [178, 179].

The antiangiogenic activity of genistein was initially detected in a study of healthy Japanese individuals who consumed a traditional soy-rich Japanese diet [180]. Urine from these subjects was collected, fractionated, and examined for activity to inhibit bFGF-stimulated endothelial cell proliferation. Of two fractions with antiendothelial activity, one contained genistein, daidzein, and *O*-desmethylangolensin. The impact of dietary soy intake was significant. In men who consumed a Japanese versus Western diet, urinary genistein was 7052 nmol/day compared to 184.4 nmol/day, respectively [181]. Soy intake has been shown to be inversely associated with cancer risk. Historically, breast cancer incidence rates have been 4 to 7 times higher among white women in the US compared to in women in China or Japan. However, when Asian women migrate to the US, their breast cancer risk rises over several generations to reach that of US white women, suggesting that modifiable factors, such as diet, rather than genetics, are responsible for the international differences. A study of over 73,000 women in China showed that daily intake of soy products such as soy milk, tofu, and fresh soybeans decreased premenopausal breast cancer risk [182]. Likewise, American women of Asian descent who consume a traditional soy-based diet have a low incidence of breast cancer [178, 183]. Early soy intake (>1.5 times per week) during childhood was found to reduce later breast cancer risk by 58% in a study of Asian women in California and Hawaii [184]. Similarly, Japanese men in Hawaii who consume a high soy diet have low mortality from prostate cancer, although the incidence at autopsy of *in situ *prostate neoplasia is similar to men in Western societies [185]. Based on laboratory findings and epidemiological data, genistein and a manufactured derivative known as genistein-concentrated polysaccharide (GCP), are being evaluated in prevention trials for prostate cancer [186].

Such clinical studies have helped dispel the theoretical concerns that soy intake may worsen breast cancer or interact with tamoxifen treatment due to the fact that genistein is a phytoestrogen. In fact, among women with breast cancer, soy food consumption has now been shown in numerous, large-scale studies to be significantly associated with decreased risk of death and recurrence, regardless of estrogen receptor status or tamoxifen use [187].

#### 4.4.3. Resveratrol

Resveratrol (3,4′,5-trihydroxystilbene) is a natural phytoalexin and polyphenol found in more than 72 plant species, such as mulberries, peanuts, grapes, and grape products, including red and rose wine. Fresh grape skins contain 50–100 *μ*g resveratrol per g and yield a concentration in Italian red wine of 1.5–3 mg/L [188]. White wine contains minimal levels of resveratrol. Resveratrol inhibits angiogenesis in the chick CAM assay, suppresses VEGF- and bFGF-induced corneal neovascularization (at 3-4 mg, equivalent to 3-4 glasses red wine/day), and inhibits tumor vascularization in T241 fibrosarcoma growing in mice [189]. Resveratrol also inhibits chemically induced mammary carcinogenesis, skin cancer tumorigenesis, and tumor growth and metastasis in mice bearing Lewis lung carcinoma [188, 189]. In mice with full thickness skin wounds, resveratrol delays wound healing angiogenesis and the time required for complete wound closure [190]. A number of antiangiogenic mechanisms have been identified, including suppression of capillary tube formation inhibition of endothelial cell DNA synthesis and binding of VEGF to human endothelial cells reduction of vascular cell adhesion molecule-1 (VCAM-1) interference with phosphorylation of endothelial mitogen-activated kinases; suppression of COX-2 enzyme and inhibition of MMP-9 expression [189, 191–193].

Red wine in particular has been repeatedly shown to have protective effects in large population studies. The California Men's Health study of over 84,000 men demonstrated an inverse relationship between red wine consumption and lung cancer incidence. Notably, there was a 61% risk reduction for men drinking at least one glass of red wine per day [194]. This protective benefit for lung cancer with greater than one glass per day was replicated in a Spanish population, using a case-control methodology involving hospitalized lung cancer patients [195]. Additionally, the Health Professionals Follow-up Study identified a protective effect for prostate cancer with a 36% risk reduction in men drinking 2–4 glasses of red wine per week [196].

#### 4.4.4. Lycopene

Lycopene, a type of natural pigment in the carotenoid family, gives tomatoes and other fruits such as watermelon and papayas their bright red color. Lycopene is an angiogenesis inhibitor which suppresses signaling by PDGF and Platelet Activation Factor *in vitro* [197]. In animal studies, lycopene suppresses spontaneous mammary tumors, hepatocarcinogenesis, colonic crypt foci, prostate cancer, and hepatoma metastases [198].

Two interventional studies have also demonstrated potent biologic effects of lycopene in the setting of malignancy. In one study, 32 men who were recently diagnosed with early-stage prostate cancer were instructed to eat one meal per day incorporating commercially made tomato sauce containing 30 mg of lycopene for 3 weeks. They then underwent curative prostatectomy; pathologic examination of the resected tissue showed a 3-fold increase of lycopene concentration in the prostate tissue, along with a slight decrease in blood prostate-specific antigen (PSA) level [199]. In a separate study, 41 men with recurrent prostate cancer were asked to consume a tomato-rich diet to achieve a minimum lycopene intake of 25 mg/day along with 40 g/day of soy protein for a total of 8 weeks. While the study design made it difficult to separate the dietary effects of lycopene from soy, it is notable that the mean serum VEGF levels of all the subjects was reduced from 87 to 51 ng/mL in a statistically significant way, and that 34% of the men experienced reduced PSA levels [200].

In the Health Professionals Follow-Up Study of over 51,000 men, the highest quintile of lycopene consumption was found to have a 15% risk reduction for developing prostate cancer compared to men in the lowest quintile [201]. The risk reduction was even greater if tomato sauce was ingested; men consuming more than 2 servings/week had a nearly 23% risk reduction compared to men consuming less than 1 serving/month.

#### 4.4.5. Omega-3 Polyunsaturated Fatty Acids (PUFAs)

Omega-3 fatty acids are unsaturated fatty acids that are vital for normal metabolism but cannot be synthesized by the human body. The best known sources of the long-chain omega-3 fatty acids—docosahexaenoic acid (DHA) and eicosapentaenoic (EPA)—are cold water oily fish such as salmon, herring, mackerel, anchovies, sardines, and trout. Both preclinical and epidemiological studies suggest that omega-3 PUFAs are effective cancer preventative agents. Omega-3 PUFAs inhibit angiogenesis by downregulating angiopoietin-2 and may competitively inhibit the bioconversion of omega-6 PUFA's into their angiogenesis-promoting derivatives such as prostaglandins and arachidonic acid [202]. In contrast, omega-6 PUFAs, present at high levels in sunflower oil, peanut oil, and corn oils, have been shown *in vitro* to stimulate endothelial migration and tube formation. Furthermore, omega-3 PUFAs have been shown to suppress Akt/m-TOR signaling pathway [203]. In animal models, Omega-3 fatty acids have been shown to suppress a variety of tumors and to prevent osteolytic metastastic lesions in bone from breast cancer [204].

Intake of omega-3 PUFAs from seafood has been associated with a decreased risk for certain cancers, including pancreatic, colon, breast, and prostate cancer. In a case-control study of 532 people diagnosed with pancreatic adenocarcinoma, consumption of omega-3 fatty acid of at least 0.85 g/day was associated with a decreased risk of pancreatic cancer; those with the highest intake had a 30% risk reduction [205]. A meta-analysis of fish intake and prostate cancer in case-control and cohort studies revealed no association between fish consumption and prostate cancer incidence, but showed a significant 63% reduction in prostate cancer mortality [206].

#### 4.4.6. Glucosinolates, Isothiocyanates, and Indole-3-carbinol

Cruciferous vegetables—which include cabbage, broccoli, cauliflower, collard greens, mustard greens, radishes, Brussel sprouts, bok choy, and kale—are rich in glucosinolates, a mustard oil glycoside that imparts a spicy, bitter flavor. The enzyme myrosinase—stored in a separate compartment of the plant cell and liberated when the plant is crushed—converts glucosinolate to the bioactive molecules isothiocyanate and indole-3-carbinol, both of which are antiangiogenic. Sulforaphane, a type of isothiocyanate, acts through inactivation of hypoxia inducible factor-1 alpha, activation of FOXO transcription factors, and promotion of endothelial cell apoptosis [207–209]. Indole-3-carbinol inhibits endothelial cell proliferation, tube formation and induces apoptosis [210, 211].

Epidemiological evidence suggests that regular dietary intake of cruciferous vegetables may lower the risk of developing several cancers. A major prospective dietary study, the European Prospective Investigation into Cancer and Nutrition (EPIC), followed the dietary habits and health of 521,468 subjects in 10 European countries between 1991–2000 [212]. After an average followup of 8.7 years, 1,830 people were diagnosed with lung cancer. Regular consumption of cauliflower and cabbage by current smokers was associated with a 23% reduction in the risk for squamous cells carcinoma of the lung. The study also found an almost 50% reduced risk of cancer of the upper digestive tract (oral cavity, pharynx, larynx, and esophagus) among people who ate the most cauliflower and cabbage (34 g/day) compared with those who ate the least (3 g/day) [213].

Another large prospective study of more than 35,000 women living in Iowa and followed for 20 years found a 18% reduced risk for non-Hodgkin's lymphoma (NHL) among women who had the highest consumption of cruciferous vegetables; in particular, consumption of at least 4 servings/month or broccoli was associated with a 28% risk reduction for non-Hodgkin's lymphoma [214]. In separate study of nearly 67,000 women in the Nurses' Health Study, more frequent dietary intake of broccoli (at least 2 servings/week) was associated with a 33% risk reduction for ovarian cancer [215].

#### 4.4.7. Flavonoids

Flavonoids are a family of polyphenols that serve as important plant pigments. Their natural roles may include acting as photoprotectants, antimicrobials, deterrents against herbivores, as well as attractants to pollinators and seed dispersant animals. They are subcategorized by chemical structure into flavones, flavonols (such as quercetin), anthocyanidins, proanthocyanidins, ellagic acid, ellagitannins, and isoflavones (such as genistein mentioned earlier), among others. In contrast, the term flav**a**nol specifically refers to the catechins, mentioned earlier. 

Flavonoids in fruits and vegetables include quercetin, anthocyanidins, proanthocyanidins, ellagitannins, among others. Flavonoids are antiangiogenic through a variety of mechanisms; they inhibit VEGF expression, inhibit endothelial cell migration, and decrease matrix metalloproteinases MMP-2 and MMP-9 [216–220]. The U.S. Department of Agriculture identifies spinach, onions, parsley, beets, and thyme among high flavonoid-containing vegetables and herbs [221]. Fresh salad greens such as lettuce, chicory, arugula, and red lettuce are also rich in polyphenolic flavonoids [222].

**Quercetin**

Quercetin is a flavonoid found in numerous types of fruits and vegetables. Its antiangiogenic properties include inhibition of MMP-2 and MMP-9 secretion from tumor cells and inhibition of endothelial cell proliferation and migration [219]. For example, quercetin has been shown to reduce *in vitro* tube formation of VEGF-stimulated human umbilical vein endothelial cells (HUVECs) grown on a 3-dimensional matrix by as much as 40% [220]. With regards to epidemiologic data, a large prospective study of 41,000 women living in Iowa between the ages of 55 and 69 found a number of flavonoid-containing leafy greens, which are abundant in quercetin, was associated with a significant risk reduction for lung cancer [223]. Women who reported eating more than six servings of leafy greens per week were nearly half as likely to develop lung cancer during the four-year follow-up period, when compared to women who ate the least greens. This benefit extended to both smokers and nonsmokers alike.

One of the richest sources of dietary flavonoids is red onion, which has particularly high levels of quercetin. Case control studies from Italian and Swiss populations have shown that moderate (1–7 servings/week) to high frequency (>7 servings/week) of onion consumption protects against a variety of cancer including colorectal, prostate, ovarian, and laryngeal cancers [224]. For example, there was a nearly 73% risk reduction for ovarian cancer in the population when comparing those who frequently consumed onion (>7 servings/week) compared to those with less frequent consumption (<1 serving/week). Similarly, there was an 88% risk reduction for esophageal cancer in the group with highest versus lowest onion intake. In a separate Dutch cohort study, consumption of at least half an onion/day was associated with 50% risk reduction in gastric cancer [225]. Finally, researchers using data from the large-scale EPIC study showed that high onion consumption was associated with a 21% risk reduction for ovarian cancer [226].

**Anthocyanins**

Anthocyanins are pigments that are present in many types of berries and grapes as well as red wine. They exhibit a purple color at neutral pH, red in acidic, and blue in alkaline conditions. Anthocyanins are end-products of the flavonoid pathway, while anthocyanidins are their aglycone precursors. Their natural function may be to attract pollinators and animals to eat the fruit or plant and disperse their seeds.

They have been shown to inhibit angiogenesis and tumor growth in experimental animals injected subcutaneously with N-nitrosomethylbenzylamine (NMBA), an esophageal carcinogen [227]. Rats that were fed an anthocyanin-rich freeze-fried extract (5% in diet) obtained from black raspberries (BRB), blackberries, or strawberries showed a reduced number of esophageal tumors as compared to controls –41% less by BRB, 46% less by blackberries, and 24% less by strawberries [228]. In another study of esophageal papillomas in rats, animals fed BRB had fewer papillomas that were of smaller volume, with reduced cell proliferation and suppression of VEGF and HIF-1alpha expression seen by immunohistochemistry as compared to the non-BRB treated mice [229, 230]. DNA microarray studies of rat esophageal carcinogenesis have shown that dietary black raspberries modulate the expression of genes associated with angiogenesis, including the cyclooxygenase and lipoxygenase pathways of arachadonic acid metabolism, as well as MMP-10 expression; CD34 staining as a marker for microvessel density was also found to be significantly reduced in the BRB diet animals [228, 229]. Berries are also rich in other natural polyphenols such as ellagic acid, which contributes to bioactivity.

An extract from black raspberries has been studied in human subjects diagnosed clinically with oral intraepithelial neoplasia. BRB 10% was applied in a gel form to the oral mucosa four times daily. After 6 weeks, there was reduced histological grade of dysplastic lesions in 50% of treated subjects, and reduced levels of COX-2 and iNOS in the lesions [231].

**Proanthocyanidins**

Proanthocyanidins are a type of tannin—large polymeric chains of flavonoids—found in many plants and fruits, notably cacao, cinnamon, cranberry, apples, grapes, black current, chokeberry, and persimmon. Proanthocyanidins are thought to be the major source of flavonoids ingested in the Western diet [232]. Historically tannins from wood bark were used as tanning agents to turn animal hide into leather due to their ability to precipitate proteins. They are also responsible for the astringent taste of certain foods and beverages [233]. Among their natural roles is protection against predation [234]. Anthocyanins and proanthocyanidins share steps in the flavonoid biosynthetic pathway. Specifically, proanthocyanidins are generated from the polymerization of flavonoid monomers, which are products of a branch pathway of anthocyanin biosynthesis.

Proanthocyanidins constitute 60% of the polyphenol content in cacao, the source of chocolate [235]. It is in the yeast-based fermentation of the raw cacao bean where the characteristic flavor precursors develop and polyphenols complex into procyanidin polymers with a core structure of (–)-epicatechin, similar to green tea [236]. Pentameric and octameric procyanidins isolated from cacao beans have been shown to inhibit the growth of cultured human aortic endothelial cells and human dermal microvascular endothelial cells (HDMECs) following angiogenic stimulation with low-level H2O2 [237]. The cacao pentameric fraction downregulates the expression of ErbB2 tyrosine kinase. Cocoa powder extract inhibits TNF-alpha-induced VEGF secretion *in vitro*. When JB6 mouse epidermal cells were pretreated with a polyphenol-rich cocoa powder extract, then exposed to TNF-alpha, there was a dose-dependent inhibition of TNF-alpha-induced VEGF expression compared to untreated controls [238]. At the highest but noncytotoxic concentration of cacao extract, VEGF expression was reduced more than twofold compared to controls.

There is evidence that cacao consumption can affect human vasculature and health. After ingestion, cacao flavonoids are stable in the gastric environment and can be detected in human plasma [239]. In studies of human volunteers, angiotensin-converting enzyme (ACE) activity is inhibited by 18% only three hours after a single intake of 75 grams of dark (72% cocoa) chocolate [240]. In a study of 4,849 individuals in Italy, those who ate one 20 gram serving of dark chocolate every 3 days had serum C-reactive protein (CRP) significantly lower than those who did not [241]. Notably, the Kuna Indians living on the San Blas Island of Panama—who have a ten-fold higher dietary intake of cacao compared to Panamanian mainlanders—have lower blood pressure, live longer, and have lower incidence of cancer compared to mainlanders [242]. Consistent with their cacao consumption, the Kuna have 6-times higher urinary excretion of cacao procyanidin metabolites than mainlanders [243].

Procyanidins are also found in cinnamon, which have been found to inhibit angiogenesis and tumor growth. Cinnamon extract inhibits VEGF receptor-2 on endothelial cells and suppresses endothelial cell proliferation, migration, and tube formation *in vitro* [244]. A water-soluble extract from dried cinnamon bark, orally administered every day or every other day for 20 days to mice bearing experimental melanomas suppresses melanoma growth [245]. The cinnamon extract suppressed tumor microvessel density, and as shown by quantitative RT-PCR, the expression of angiogenic factors VEGF, FGF, and TGF-*β* as well as COX-2 and HIF-1alpha, which promote angiogenesis. In addition, the cinnamon extract suppresses melanoma metastasis as measured by the size and weight of the spleen and draining lymph nodes of mice bearing melanoma *in vivo* [245].

Many edible berries also contain proanthocyanidins, including the American cranberry, black currants, and grapes. Chokeberries, named because of their extreme astringency, contain among the highest levels of proanthocyanidins detected [246]. Mixtures of berry extracts inhibit tube formation of endothelial cells in Matrigel [247]. Extracts from blueberries, bilberries, raspberries, and strawberries suppress VEGF expression by immortalized human keratinocytes (HaCaT cells) *in vitro* and this VEGF inhibition appears to be independent of the antioxidant property of the extracts [247]. Experimental liver cancer in rodents induced by the carcinogen DENA are suppressed in number and size by an extract from black currants added to the animal's diet (equivalent to 500 mg/kg body weight) [248]. Procyanidins in cranberries may have multiple health benefits including both chemopreventive properties as well as maintaining bladder health due to inhibiting *E. Coli* adherence to uroepithelium [249]. A cranberry extract tested on DU145 human prostate cancer cells significantly inhibited expression of MMP-2 and MMP-9 and increased expression of TIMP-2 *in vitro*, consistent with mechanisms that suppress angiogenesis [250]. Cranberry juice, given as a 20% solution, reduces the incidence of azoxymethane-induced colonic aberrant crypts in rats by 77% versus drinking water controls when supplemented for three weeks before and ten weeks after carcinogen exposure [251].

Both apples and apple juice are rich sources of procyanidins in addition to other polyphenols previously described such as quercetin and catechins. Based on the USDA Continuing Survey of Food Intakes by Individuals (CSFII), apples are a major source of proanthocyanidins in the U.S. diet [232]. In a recent study, scientists examined the specific chemopreventive properties of a polyphenol extract of apple juice. Apple procyanidins were found to inhibit Cox-1 [252]. Several studies have shown that cloudy apple juices, such as apple cider, contain much higher concentrations of procyanidins than clear apple juices. Specifically, one study determined that the suspended particles in cloudy apple juice contain up to 60% of apple procyanidins in the juice.

Epidemiological studies have provided accumulating evidence that apples have cancer-preventive properties, particularly against lung and colorectal cancers. In the Nurses Health Study involving 77,000 women, a statistically significant 37% risk reduction for lung cancer was observed among women for increases of 1 serving per day of apples or pears [253]. Similar results were obtained from a Finnish cohort study involving 10,000 men and women [254]. The results of a case-control study conducted in Hawaii with 528 lung cancer cases and 528 controls found a statistically significant decrease in lung cancer risk with increased consumption of apples [255]. An analysis of case-control studies conducted in Italy found that people who consumed at least one apple per day had a significantly reduced risk of colorectal cancer and cancers of the oral cavity, larynx, breast, and ovary relative to those who ate less than an apple a day [256]. In a prospective cohort of 35,159 Iowa women aged 55–69 years, intake of apple juice or cider was associated with lower risk of developing non-Hodgkin's lymphoma [214].

**Ellagitannins**

Ellagitannins are glycosides of the flavonoid ellagic acid and fall under the category of hydrolyzable tannins. They can be found in numerous types of fruits and nuts including pomegranate, strawberries, blackberries, raspberries, muscadine grapes, walnuts, and pecans [257]. Upon consumption, ellagitannins are hydrolyzed to ellagic acid which is antiangiogenic. Colonic microbiota further convert ellagic acid to urolithin A which is also bioactive [258].

All parts of the pomegranate contain high levels of ellagitannins, of which the primary type is punicalagin. Bioactive polyphenols are found not only in the edible aril (pulp and seed) which is popularly consumed, but also concentrated in the peel of the fruit, which is not usually consumed but may be part of the juice extraction by some processors. Indeed, polyphenol extraction increases 6.5-fold when the whole fruit is processed compared to juice from arils alone [259]. Ellagitannins have been shown to inhibit the growth of prostate cancer in both *in vitro* and *in vivo *laboratory experiments, and studies have suggested that the compound's antiangiogenic properties play a role in this inhibition. Interestingly, pure pomegranate juice has been found to be more potent than its separated individual polyphenol components, likely due to synergistic effects [260]. Effects of pomegranate extract (POMx)—derived from the skin and seeds of the pomegranate fruit—have been shown on endothelial cells (HUVECs) and human prostate cancer cells (LNCaP line) *in vitro* [261]. Pomegranate extract inhibited the proliferation of both cell types and suppressed the secretion of VEGF and HIF-1alpha. Mice implanted with human prostate tumors derived from the LAPC4 tumor line were administered the human equivalent of 1.7 cups (8 oz) of pomegranate juice per day. Pomegranate juice significantly decreased tumor microvessel density, as well as tumor size, compared to the control animals [261]. Pomegranate juice, when given as a 20% solution, reduces the incidence of azoxymethane-induced colonic aberrant crypts in rats [251]. In a study of men with recurrent prostate cancer who drank 1 cup of pomegranate juice per day, their prostate-specific antigen (PSA) doubling time was extended from 15 to 54 months [262].

#### 4.4.8. Menaquinone

A form of vitamin K, menaquinone (vitamin K2), found in certain food sources is antiangiogenic and associated with a reduced risk for developing several forms of cancer. Menaquinone is distinct from the phylloquinone (Vitamin K1) present in dark leafy vegetables. Instead, menaquinone is a fat—soluble vitamin formed naturally by bacteria in fermented dairy products, including cheese and yogurt, in fermented soy such as natto, and also present in dark meat. Certain cheeses, such as Dutch Gouda, Swiss Emmental, and Norwegian Jarlsberg, have particularly high concentrations of menaquinone. In laboratory studies, menaquinones suppress angiogenesis, enhance tumor apoptosis, and inhibit the proliferation of cancer cells [263].

A subpopulation of the European Prospective Investigation into Cancer and Nutrition (EPIC) study, called the Heidelberg cohort, followed the diet and health status of more than 24,300 participants for at least ten years, starting in 1994. The participants answered detailed questionnaires at regular intervals about their diet and general health. During the follow-up period, 1,775 cancer cases were diagnosed, of which 458 were fatal. Among the foods documented, participants who consumed the most cheese (at least 41 g/day) had a significantly reduced risk of dying from cancer compared with those who consumed the least (less than 14 g/day) [264]. Cheese consumption contributed to about 45% of total menaquinone intake. In terms of specific cancers, higher consumption food containing menaquinone was associated with significantly lower incidences of lung cancer and prostate cancer. Participants with the highest levels of menaquinone in their diets had a 62% reduced risk of lung cancer compared with those with the least, and a similar reduced risk of dying from lung cancer. 

Prospective clinical studies of vitamin K2 have shown chemopreventive activity in patients. In a study of 40 women in Osaka diagnosed with viral liver cirrhosis, individuals were assigned randomly to an intervention or control group. During more than 7 years of followup, the cumulative proportion of people who developed hepatocellular carcinoma (HCC) was significantly smaller in the group that received vitamin K2 (45 mg/day) [265]. Vitamin K2 decreased the risk of HCC to 20% compared to that of the control group. On an annual incidence basis, HCC developed in only 1.6% in the treatment group compared with 8.8% in the control group and 7.9% in the general cirrhotic population.

#### 4.4.9. Curcumin

Curcumin (diferuloylmethane), a flavonoid derived from the plant *Curcuma longa*, is present in tumeric spice. It has chemopreventive and antiangiogenic activity and inhibits carcinogenesis in skin, stomach, intestines, and liver. Dietary ingestion of curcumin has been shown to prevent the formation of colon polyps, suppress proliferation of colon cancer and prostate cancer cells, and decrease intratumoral microvessel density [266–268]. Studies of endothelial cells exposed *in vitro* to curcumin show induction of apoptosis; downregulation of gene transcripts for VEGF, bFGF, and MMP-2; COX-2 inhibition; upregulation of TIMP; disruption of vascular tube formation; and inhibition of endothelial cell motility by interfering with the Ras-mediated c-Jun N-terminal kinase (JNK) pathway [269–273]. 

A Phase I prevention trial of curcumin (500–800 mg/day) showed histological improvement of lesions in patients with various malignant and premalignant lesions, including recently resected bladder cancer, oral leukoplakia, intestinal metaplasia, CIN, and Bowen's disease [274]. In a Phase II study, dietary curcumin was given for 30 days to 44 subjects with aberrant crypt foci (ACF), a premalignant marker for colorectal cancer. Those who consumed 4 g/day of curcumin had a 40% reduction in the number of rectal ACF lesions [275]. 

#### 4.4.10. Beta-cryptoxanthin

Beta-cryptoxanthin is a natural carotenoid pigment present in brightly colored orange, red, or yellow foods. Structurally it is related to beta-carotene, and is ultimately metabolized to vitamin A in the body and can be found circulating in the blood after consuming carotenoid-rich foods. Vitamin A and retinoic acid analogs are antiangiogenic and synthetic derivatives have been investigated as chemopreventive agents [276–280]. 

Papaya is a rich source of carotenoids, with the same lycopene content as tomatoes, but twice the beta-cryptoxanthin content. Studies in tropical populations have shown that increased daily consumption of papayas results in higher levels of beta-cryptoxanthin in the blood [281]. Consumption of such beta-cryptoxanthin-rich foods may reduce cancer rates in high-risk populations. For example, higher papaya consumption has been shown to be inversely associated with the risk of developing high-grade cervical lesions. A nested case control study involving a population of HPV-positive women in Brazil showed that consuming one or more servings of papaya per week cut the risk of developing a high grade cervical lesion by 81% [282].

Other studies have shown a risk reduction with the consumption of papaya and carotenoid-rich fruits for other cancers, including lung and gall bladder cancers [283, 284]. In the Singapore Chinese Health Study, 63,257 Chinese men and women ages 45–74 participated in a prospective study of diet and cancer [284]. Using a food composition database, an estimate of their carotenoid intake, including beta-cryptoxanthin was quantified. In the first 8 years of follow-up, 482 lung cancer cases occurred in the cohort. A high level of dietary beta-cryptoxanthin was associated with reduced risk of lung cancer. Comparing the highest to lowest quartile, there was a 27% risk reduction among all subjects, and a 37% risk reduction among current smokers.

In a study of women, diet, and breast cancer risk, 403 breast cancer cases and 602 controls from the Nurses' Health Study were examined for high breast densities, a strong predictor for breast cancer risk [285]. Overall, circulating total carotenoids were inversely associated with breast cancer risk. Among women in the highest tertile of mammographic density, total carotenoids were associated with a 50% reduction in breast cancer risk. 

#### 4.4.11. Other Novel Dietary Inhibitors

We have identified antiangiogenic activity in a number of other dietary-derived chemopreventive molecules. These include brassinin, a phytoalexin found in Chinese cabbage; the citrus-derived bioflavonoids hesperidin and naringenin; ellagic acid from berries, pomegranate, and grapes; silymarin from milk thistle and artichoke; and the organosulfur allyl disulfide derived from garlic [286–291]. Further studies are underway to define their molecular targets in angiogenesis, their optimal biological doses, and efficacy in inhibiting tumor vascularity.

The diverse natural sources of these and other antiangiogenic chemopreventive molecules raise the possibility of designing scientific diets for patients at high risk for cancer, or for those with known disease to chronically suppress angiogenesis and tumorigenesis.

## 5. Conclusion and Future Directions

Angiogenesis is a critical, rate-limiting step in the development of all known cancers, and its inhibition suppresses tumor growth, progression, and metastases. Antiangiogenic therapy represents a new approach to the early intervention and prevention of malignant disease. During the next two decades, the total yearly number of newly diagnosed cases of cancer is projected to rise from 12.4 million new cases per year in 2008 to 26.4 million in 2030, and the number of annual deaths is projected to increase 170%, to 17 million [292, 293]. According to the World Health Organization and International Agency for Research on Cancer, cancer is the leading cause of death worldwide as of 2010 [293]. The implementation of effective chemoprevention strategies based on angiogenesis inhibition attained through dietary sources may decrease these numbers in a cost-effective and quality of life enhancing manner. Dietary effects are already thought to underlie many of the large international differences in incidence seen for most cancers [294]. Indeed, nutritional factors have been estimated to contribute to 20–60% of cancers worldwide and to approximately one-third of deaths from cancer in Western countries [295]. 

The identification of dietary sources of antiangiogenic molecules has been aided tremendously through observational epidemiologic studies, which have the ability to identify specific foods associated with reduced cancer risk. These studies generally fall under the categories of prospective cohort and retrospective case-control studies, and more of both of these types of studies will be necessary to identify additional sources of antiangiogenic compounds and to help confirm the chemopreventive properties of previously identified foods and their constituent bioactive molecules. 

The dietary prospective cohort study allows thorough assessment of dietary exposures with a reduced risk of recall bias as patients are initially cancer-free when enrolled. As study participants are followed into the future and observed for the development of cancer and other cancer-related endpoints, investigators can identify specific foods present in participants' diets that are associated with cancer risk reduction. With the evolving understanding of the underlying molecular basis of various naturally antiangiogenic foods, an important consideration for future cancer prevention cohort studies will be to ensure accurate assessment of participants' dietary intake of bioactive compounds. This will include not only comprehensively querying participants about all potential dietary sources of the bioactive compounds of interest, but also obtaining more detailed information about the preparation of foods, as this can significantly impact the amount of antiangiogenic molecules obtained through diet. Furthermore, the subtypes of foods consumed, such as the specific variety of apple or tomato eaten by a participant, and the way the food is processed or cooked prior to consumption, can strongly affect the amount of consumed bioactive molecules and would be important information to collect. Thus, while the food frequency questionnaires (FFQs) used in past cohort studies have attempted to quantify the intake of various foods, future studies focused on antiangiogenesis may be able to make a more detailed and accurate assessment of this particular aspect of dietary intake. 

Case-control studies can also be valuable as a method for expediently identifying promising dietary exposures without the need for the long timeframes and massive study populations that typically characterize dietary cohort studies. While case-control studies are nearly always vulnerable to recall bias on the part of the cancer patients who compose the case populations, they are a valuable starting point in identifying promising risk-reducing foods that can be further explored through prospective and, more recently, interventional dietary studies. The key to these studies will be the use of well-designed dietary survey instruments and methods to allow data capture on short time horizons that can accurately gauge the intake of antiangiogenic and chemopreventive biomolecules. Finally, interventional dietary studies are a particularly promising methodology with which to confirm the antiangiogenic and chemopreventive properties of specific foods and bioactive molecules. In particular, recently diagnosed cancer patients or those with pre-invasive angiogenesis-dependent lesions such as colonic adenomas, prostatic intraepithelial neoplasia, cervical intraepithelial neoplasia, and actinic keratoses could potentially show significant benefit from short-term antiangiogenic dietary interventions with regards to progression, metastasis, or recurrence of their lesions [167]. Investigators with access to serum or biopsy samples could also follow changes to angiogenesis biomarkers, tumor biomarkers, or tumor characteristics [262, 296]. Because of their experimental nature, randomized interventional dietary studies have the potential to become a valuable method for validating the antiangiogenic nature of bioactive molecules and foods. 

The United States Department of Agriculture's update to its longstanding *Food Pyramid* dietary recommendations—namely, its replacement with the new *MyPlate* initiative that pushes for an increase in the amount of whole fruits and vegetables consumed at each meal—does appear to indirectly promote increased consumption of certain foods that contain natural sources of antiangiogenic molecules [297]. For example, specific recommendations of the initiative include glucosinolate-rich cruciferous vegetables, lycopene-containing tomatoes, resveratrol-bearing grape products, and beta-cryptoxanthin-abundant orange and yellow vegetables.

Data and conclusions from rigorous dietary cancer prevention studies should be implemented into public health policy. Optimal health outcomes result not just from high quality medical care, but also from diet and lifestyle patterns that can intercept disease at the earliest microscopic stages. Investments in cancer prevention, guided by the biological principles of antiangiogenic therapy, will expand on the substantial clinical applications already established by the biopharmaceutical industry. Cancer prevention using antiangiogenic factors present in widely available foods further offers an egalitarian strategy for large populations in societies that increasingly recognize the value of health-promoting dietary choices. Many dietary sources of angiogenesis inhibitors are common ingredients in the world's most popular culinary traditions (i.e., Asian, Mediterranean, etc.). We propose that an antiangiogenic diet is a practical and cost-effective method to reduce the risk of cancer and other diseases and to enhance quality of life.

In summary, tumor angiogenesis is a critical target for cancer prevention. Natural antiangiogenic molecules are present in numerous dietary sources and represent a wide spectrum of mechanisms that can suppress the growth of microscopic tumors. The control of blood vessel growth through dietary antiangiogenesis promises to redefine cancer as a disease that can be suppressed throughout an individual's lifetime, from infancy through adulthood.

## Figures and Tables

**Table 1:** Angiogenic factors.

Angiogenin	
Angiopoietin-1	
Adrenomedullin	
Del-1	
Fibroblast growth factor-1 (acidic FGF, FGF1)	
Fibroblast growth factor-2 (basic FGF, FGF2)	
Follistatin	
Granulocyte-colony-stimulating factor (G-CSF)	
Hepatocyte growth factor/scatter factor (HGF/SF)	
Interleukin-3 (IL-3)	
Interleukin-8 (IL-8)	
Intermedin	
Keratinocyte growth factor (FGF-7)	
Leptin	
Midkine	
Neuregulin	
Osteogenic protein-1	
Placental growth factor (PlGF)	
Platelet-derived endothelial-cell growth factor (PD-ECGF)	
Platelet-derived growth factor (PDGF)	
Pleiotrophin	
Progranulin	
Proliferin	
Transforming growth factor-*α* (TGF*α*)	
Transforming growth factor-*β* (TGF*β*)	
Tumor necrosis factor-*α* (TNF*α*)	
Vascular endothelial growth factor/vascular permeability	
factor (VEGF/VPF)	

**Table 2:** Endogenous inhibitors of angiogenesis.

Angiopoietin-2 (in the absence of VEGF)	
Angiostatin	
Antithrombin III fragment	
Arresten	
Canstatin	
Chondromodulin I	
Connective tissue growth factor (CTGF)	
Decorin	
Endorepellin	
Endostatin	
Fibronectin 20-kDa fragment	
Interferons-*α*, *β*, and *γ*	
Interleukin-4 (IL-4)	
Interleukin-10 (IL-10)	
Interleukin-12 (IL-12)	
Interferon-inducible protein-10 (IP-10)	
Kringle 5	
Metastatin	
METH-1	
METH-2	
2-Methoxyestradiol	
Osteopontin cleavage product	
PEX	
Pigment epithelium-derived factor (PEDF)	
Plasminogen activator inhibitor (PAI)	
Platelet factor-4	
Prolactin 16-KDa fragment	
Proliferin-related protein	
Prothrombin kringle 2	
Maspin	
Restin	
Soluble fms-like tyrosine kinase-1 (S-Flt-1)	
SPARC cleavage product	
Tetrahydrocortisol-S	
Tissue inhibitors of matrix metalloproteinases (TIMPs)	
Thrombospondin-1 and -2	
Transforming growth factor-*β* (TGF-*β*) (*activated form) *	
Troponin-1	
Tumstatin	
Vascular endothelial growth inhibitor (VEGI)	
Vasostatin	

**Table 3:** Genetic control of angiogenesis.

Id1 p53	
Id3 Rb	
HIF-1a VHL	
K-*ras *PTEN	
N-*myc trk *B	
c-*myc *p16INK4a	
c-*fos *	
c-*src *	
c-*myb *	
c-*jun *	
HER2/*neu *	
EGFT	
Raf	
Mek	
p73	
Del-1	
FzD	
Bcl2	
MDNM2	
PML-RAR	
ElF-4E	

**Table 4:** Approved antiangiogenic agents and cancer indications.

Bevacizumab (Genentech/Roche)	*Colon, Lung, Breast, Brain, Kidney*
Cetuximab (Bristol-Myers Squibb/Imclone)	*Colon, Head and Neck*
Endostatin (Simcere)^†^	*Lung*
Erlotinib (Genentech/Roche/OSI)	*Lung, Pancreatic*
Everolimus (Novartis)	Kidney, Pancreatic/NET*, Brain/SEGA**
Imiquimod (Graceway/3M)	*Actinic keratosis, Basal cell carcinoma*
Interferon alfa (Roche/Schering)	*Melanoma, Kaposi's sarcoma*
Lenalidomide (Celgene)	*Myleodysplastic syndrome, Multiple myeloma*
Pazopanib (GlaxoSmithKline)	*Kidney*
Sorafenib (Bayer/Onyx)	*Kidney, Liver*
Sunitinib (Pfizer)	Kidney, GIST, Pancreatic/NET*
Temsirolimus (Wyeth)	*Kidney, Lymphoma*
Thalidomide (Celgene)	*Multiple myeloma*
Vandetanib (AstraZeneca)	*Thyroid*

^†^Available only in China.

*Neuroendocrine tumor.

**Subependymal giant cell astrocytoma, associated with tuberous sclerosis.

Source: Angiotracker, The Angiogenesis Foundation (http://www.angio.org/).

**Table 5:** Chemopreventive agents that possess antiangiogenic properties.

Alpha-difluoromethylornithine (DFMO)	
Aspirin	
Brassinin	
Celecoxib	
Curcumin	
1 *α*,25-dihydroxyvitamin D3	
Ellagic acid	
Epigallocatechin 3-gallate	
Finisteride	
Genistein	
*N*-acetylcysteine (NAC)	
Naringenin	
Oltipraz	
Resveratrol	
Retinoids	
Selenium	
Silymarin	
Statins	
Sulindac	
Tamoxifen	

Source: Angiotracker, The Angiogenesis Foundation (http://www.angio.org/).
